# Factors associated with restenosis after carotid artery stenting differ between closed-cell and open-cell stents

**DOI:** 10.3389/fneur.2025.1726023

**Published:** 2026-01-06

**Authors:** Yayue Liu, Yujie Sun, Guangwen Li, Zunwei Wang, Yan Sun, Yong Zhang

**Affiliations:** 1 Qingdao University, Qingdao, Shandong, China; 2 Department of Neurology, The Affiliated Hospital of Qingdao University, Qingdao, Shandong, China

**Keywords:** carotid, carotid stenosis, closed-cell, open-cell, restenosis, stent design, stroke

## Abstract

**Background:**

There is currently a lack of research comparing factors associated with in-stent restenosis (ISR) after carotid artery stenting (CAS) between closed-cell stents (CCS) and open-cell stents (OCS). The objective of this study was to assess and compare ISR incidence and clinical outcomes in CCS and OCS cohorts, proposing that ISR-related risk factors may differ based on stent design.

**Materials and methods:**

We retrospectively analyzed clinical data from patients who underwent CAS at our institution between 2020 and 2023. The median follow-up was 15.0 months, ranging from 11.0 to 23.0 months, with the longest follow-up period being 61 months. Patients with less than 6 months of follow-up were excluded from analysis. ISR was identified by either a peak systolic velocity (PSV) exceeding 300 cm/s or stenosis equal to or greater than 50%, as evaluated by CTA or DSA. Risk factors linked to ISR within CCS and OCS groups were initially examined through univariate Cox regression analysis. Multivariate Cox regression models were developed by employing stepwise regression and backward elimination methods.

**Results:**

This study included a total of 257 CAS procedures, comprising 129 CCS-treated patients and 128 OCS-treated patients. No significant differences were found between groups in demographic factors, comorbid conditions, or perioperative and follow-up clinical outcomes. Compared with the OCS group, the CCS cohort demonstrated significantly greater preoperative stenosis, higher frequency of post-dilation interventions, and lower residual stenosis rates (all *p* < 0.05). Despite these differences, ISR rates between the two groups were not statistically significant (log-rank *p* = 0.073). Patients who experienced ISR exhibited a notably greater degree of preoperative stenosis (*p* = 0.039). Within the CCS cohort, symptomatic atherosclerotic stenosis was significantly related to ISR (*p* = 0.038). Conversely, in the OCS cohort, significant predictors of ISR included residual stenosis severity and the presence of diabetes mellitus (both *p* < 0.05).

**Conclusion:**

Although initial analysis indicated no statistically significant ISR differences between CCS and OCS groups, multivariate regression analyses highlighted distinct independent risk factors for ISR based on stent type. Specifically, symptomatic atherosclerotic stenosis emerged as a significant risk factor in CCS-treated patients, while residual stenosis severity and diabetes mellitus were principal risk factors in the OCS-treated group.

## 1 Introduction

CAS serves as an alternative therapeutic strategy to carotid endarterectomy for patients presenting with carotid stenosis who either are unsuitable for open surgery or have elevated complication risks (1), particularly those younger than 70 years of age (2). The Carotid Revascularization Endarterectomy versus Stenting Trial (CREST) revealed no significant differences in long-term primary endpoints between CAS and endarterectomy groups (3). CCS and OCS are utilized based on distinct clinical indications; however, prior studies yield conflicting findings regarding ISR rates between these two stent designs.

Many studies have suggested superior outcomes associated with OCS over CCS. A meta-analysis demonstrated significantly lower restenosis rates in OCS groups, with thresholds of ≥40% stenosis (OR = 0.42, 95% CI 0.19–0.92; I2 = 0%) and ≥70% stenosis (OR = 0.23, 95% CI 0.10–0.52; I2 = 0%) (4). These analyses included studies published in English before October 31, 2017, employing diverse imaging modalities to evaluate ISR. Müller et al. (5), in the International Carotid Stenting Study (ICSS), similarly indicated reduced restenosis risk in OCS-treated patients, defining stenosis severity based on ultrasound velocities of >1.3 m/s for ≥50% and >2.1 m/s for ≥70% stenosis. Another recent study from 2021 validated these results utilizing carotid duplex ultrasound (6). In 2025, Polania-Sandoval et al. (7) reported a significantly higher restenosis rate with CCS (*p* < 0.001), including both transfemoral CAS and trans-carotid artery revascularization (TCAR). However, CCS may have higher PSV (5).

Other studies, however, have shown opposite findings. Alparslan et al. (8) reported significant differences between stent types on imaging, suggesting more intimal hyperplasia development with OCS. Lai et al. (9) found OCS had a higher restenosis rate after 738 CAS procedures, defining ISR as >70% stenosis on digital subtraction angiography (DSA).

Clinicians should consider differences in clinical applications caused by the mechanical properties of these two stent types. CCS has smaller cell areas, which provide better luminal support and retain atherosclerotic debris. A study from the CREST-2 Registry (C2R) reported that CCS was associated with lower periprocedural stroke or death rates (10). High-risk vascular anatomy significantly correlates with periprocedural complications (11). Therefore, CCS may better tolerate post-dilation when residual stenosis remains unsatisfactory, as plaque disruption occurs during the procedure. Higher radial force also helps reduce residual stenosis. Previous studies reported that lower residual stenosis after extracranial carotid stenting is protective against ISR (12).

OCS, however, has superior flexibility and vessel wall adherence. Advantages in tortuous vessels or bifurcation lesions have been reported, including reduced risk of perioperative complications and restenosis in cases with specific anatomical characteristics (13, 14). One study suggested that OCS is preferred in highly tortuous anatomy (10). Another reported that OCS was associated with lower odds of in-hospital stroke/death when used across the carotid bifurcation, possibly due to improved conformity to varying vessel diameters (13). Mechanical irritation and inflammatory responses may induce intimal hyperplasia (15). Advancing research allows interventionalists to consider these benefits and risks when selecting stent types, helping them choose appropriate devices to reduce perioperative complications and restenosis. Previous studies identified individual factors (age, obesity, diabetes, hypertension, dyslipidemia, smoking), lesion factors (stenosis severity, plaque calcification, lesion length, contralateral carotid stenosis), and surgical factors (stent design, residual stenosis) as risk factors associated with ISR (9, 15–18). Comparative studies analyzing ISR risk factors between the two stent types remain scarce.

This study aims to compare ISR rates and clinical outcomes between CCS and OCS, hypothesizing that ISR risk factors (individual, lesion, and surgical factors) differ according to stent design.

## 2 Materials and methods

### 2.1 Patients and materials

This retrospective analysis incorporated data from 257 CAS procedures conducted at our institution between 2020 and 2023.

Inclusion criteria comprised: (1) patients aged 18–80 years; (2) asymptomatic carotid stenosis ≥70% or symptomatic atherosclerotic stenosis ≥50%, adhering to NASCET criteria; (3) availability of imaging follow-up for a minimum of 6 months post-surgery. Exclusion criteria included: (1) carotid stenosis not resulting from atherosclerosis, such as arterial dissection, Moyamoya disease, or arteritis; and (2) missing follow-up or procedural DSA imaging data.

Patients received dual antiplatelet treatment (aspirin 100 mg daily at night and clopidogrel 75 mg daily) for at least 5 days preoperatively and 3 months postoperatively. Subsequently, antiplatelet therapy continued as aspirin (100 mg nightly) or clopidogrel (75 mg daily). All patients also underwent long-term statin therapy for lipid control. CCS included Wallstent (Boston Scientific), while OCS included Acculink (Abbott) and Precise (Cordis). At our institution, CCS were preferred for symptomatic stenosis, particularly for lesions with a high embolic risk. For vessels with significant curvature or angulation, open-cell stents (OCS) were favored. For lesions with significant ulceration, CCS were personally preferred over OCS. For stenting calcified lesions, CCS with post-dilation were recommended.

Ethical approval for this retrospective study was granted by the Institutional Ethics Committee (Approval Number: QYFYWZLC30400).

### 2.2 Data collection and follow-up

Data collected included demographics, comorbidities, surgical details, DSA imaging, and clinical follow-up information. Recorded comorbidities included diabetes mellitus, previous stroke events, coronary artery disease (CAD), and hypertension. Smoking status was categorized into never-smokers, former smokers, or current smokers. Surgical parameters documented comprised stent type, dimensions (diameter and length), and pre- or post-dilation procedures. Endpoint events were recorded as the initial detection of ISR or the last negative imaging result due to variable patient compliance.

The same imaging protocol was applied to both CCS and OCS group. Immediate post-stenting residual stenosis was measured uniformly using DSA in all patients. Follow-ups at 6 months postoperatively and annually thereafter were required. Patients 6 months or more after surgery require at least carotid vascular ultrasonography screening and were advised to undergo further imaging studies, especially who exhibiting significantly accelerated blood flow. Restenosis was defined as stenosis ≥50% occurring within the stent or within 5 mm of either end according to NASCET criteria (19). CTA-based assessments were only performed during follow-up when DSA was not available. In patients for whom CTA was the highest-level imaging modality available, stenosis severity was assessed using NASCET criteria. All vascular imaging interpretations were performed independently by two experienced interventional neuroradiologists.

Symptomatic atherosclerotic stenosis was defined as stroke, TIA, or transient monocular blindness ipsilateral to the stenosis within 6 months before intervention (20). Symptomatic in-stent restenosis was defined as restenosis associated with TIA or stroke occurring in the previously treated vessel segment (21).

Clinical follow-up outcomes recorded were stroke, hemorrhagic events, and death. Stroke was characterized by new neurological deficits persisting for over 24 h (4). Hemodynamic depression (HD) was defined as sustained hypotension or bradycardia during or immediately following the procedure (22).

### 2.3 Statistical analysis

Continuous variables underwent normality assessment using the Shapiro–Wilk test and were presented as mean ± SD or median (interquartile range). Group comparisons utilized Student’s *t*-test or the Mann–Whitney *U* test. Categorical data were expressed as proportions and compared using chi-square or Fisher’s exact tests. To identify ISR-associated risk factors in both CCS and OCS cohorts, univariate Cox regression analyses were conducted. Considering the small sample size, variables with significance levels close to 0.1 may also be included to reasonably reflect the potential influence. Variables with *p*-values <0.1 from univariate analyses and clinically plausible variables regardless of significance progressed to multivariate analysis. Age (12) and residual stenosis (12, 17, 23) were also included in the multivariate model for OCS based on previous research implications. Stepwise regression and backward elimination methods facilitated variable selection for multivariate Cox models. An additional multivariate Cox regression was conducted for adjustment because of baseline stenosis severity imbalance, incorporating stent type and preoperative stenosis grade. Statistical significance was set at *p* < 0.05, and analyses were performed using R software (version 4.4.2).

## 3 Results

**Figure 1:**
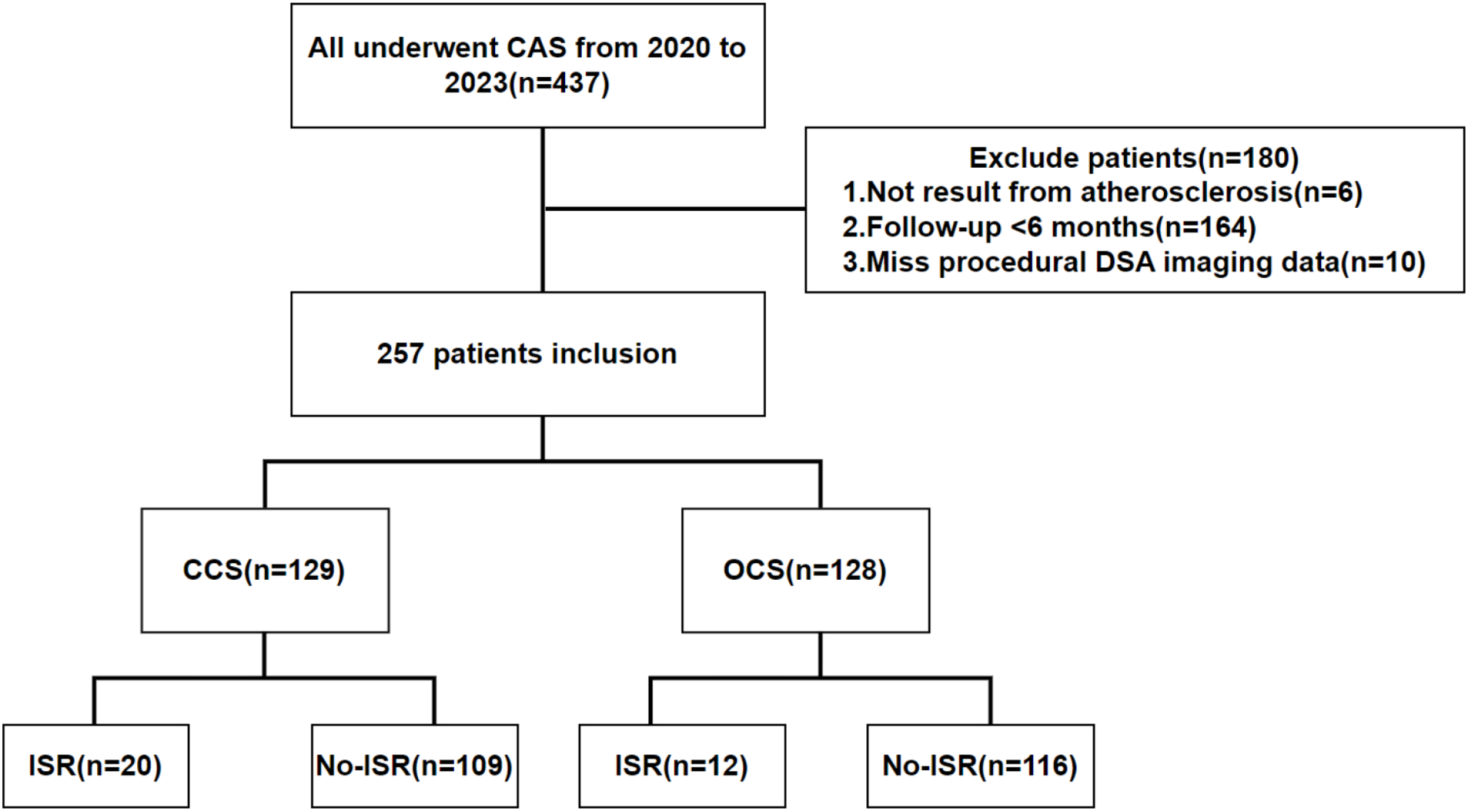
Study flowchart. CAS, carotid artery stenting; DSA, digital subtraction angiography; CCS, closed-cell stent; OCS, closed-cell stent; ISR, in-stent restenosis.

This study encompassed a total of 257 CAS procedures (Figure 1), involving patients with a median age of 67.0 years (interquartile range, 62.5–71.0 years). Among the cohort, males accounted for 221 cases (86.0%), and symptomatic atherosclerotic stenosis was present in 116 individuals (45.1%). Specifically, 129 cases received CCS, while 128 underwent treatment with OCS. Median follow-up via imaging was 15.0 months (interquartile range, 11.0–23.0 months), extending up to 61 months in certain cases. ISR occurred in 32 patients (12.5%), with 20 cases (15.5%) in the CCS subgroup and 12 cases (9.4%) in the OCS subgroup. Only two patients declined imaging due to personal reasons; their target vessel velocities were both ≥300 cm/s. We concluded that restenosis was evident in these cases.

### 3.1 Comparison between patients with and without ISR

No significant differences emerged regarding demographic parameters or existing comorbidities between ISR-positive and ISR-negative patients (Table 1). Nevertheless, preoperative stenosis severity was significantly higher in ISR-positive patients (*p* = 0.027).

**Table 1:** Comparison between patients with and without ISR.

Variable	ISR−(*N* = 225)	ISR+(*N* = 32)	*p*
Demographics			
Age (years)	67.0 (63.0, 71.0)	68.0 (61.0, 72.0)	0.935b
Sex (male)	191 (84.9%)	30 (93.8%)	0.281**
Comorbidities			
Diabetes mellitus	67 (29.8%)	13 (40.6%)	0.215
CAD	58 (25.8%)	9 (28.1%)	0.777
Hypertension	165 (73.3%)	23 (71.9%)	0.862
Surgical details			
Stent style (CCS)	109 (48.4%)	20 (62.5%)	0.137
Preoperative stenosis (%)	75.2 ± 12.7	80.4 ± 10.3	**0.027a**
Residual stenosis (%)	20.5 (7.6, 28.4)	22.1 (10.7, 28.2)	0.501b
Symptomatic atherosclerotic stenosis	98 (43.6%)	18 (56.3%)	0.177
Periprocedural clinical event			
HD	7 (3.1%)	1 (3.1%)	1.000**
Stroke	3 (1.3%)	0 (0.0%)	1.000*
Any hemorrhage	5 (2.2%)	1 (3.1%)	0.553*
Follow-up clinical event			
Stroke	21 (9.3%)	13 (40.6%)	<0.001**
Ipsilateral stroke	6 (2.7%)	8 (25.0%)	<0.001**
Any hemorrhage	2 (0.9%)	2 (6.3%)	0.077*

Results are presented as numbers and percentages or as mean ± standard deviations or median (interquartile ranges). LDL, low-density lipoprotein cholesterol; BMI, body mass index; CAD, coronary heart disease; TIA, transient ischemic attack; HD, Hemodynamic depression; mRS, Modified Rankin Scale; ISR, in-stent restenosis; CCS, closed-cell stent; symptom-treatment: days from symptom to CAS procedure. The values with bold type represent statistically significant results with a *p*-value <0.05.

*Fisher exact test; **continuity correction. a. Student’s *t*-test; b. Mann–Whitney U test.

No notable variations were detected between ISR and non-ISR groups in periprocedural clinical outcomes, including HD (*p* = 1.000), stroke (*p* = 1.000), or hemorrhage events (*p* = 0.553).

Throughout the follow-up period, stroke and ipsilateral stroke incidences were significantly lower in the non-ISR group (both *p* < 0.001). Conversely, no significant differences in hemorrhagic complications emerged during follow-up (*p* = 0.077).

### 3.2 Comparison of patient characteristics between CCS and OCS groups

Comparative analysis between CCS and OCS groups revealed no significant differences in demographics or comorbidities (Table 2). However, patients treated with CCS exhibited greater preoperative stenosis (*p* = 0.041), increased frequency of post-dilation procedures (*p* < 0.001), and less residual stenosis post-procedure (*p* = 0.029). No statistically significant differences in periprocedural events, such as HD (*p* = 0.276), stroke (*p* = 0.995), or hemorrhage (*p* = 0.687), were observed.

**Table 2:** Comparison of patient characteristics between CCS and OCS groups.

Variable	Closed-cell stent (*N* = 129)	Open-cell stent (*N* = 128)	*p*
Demographics			
Age (years)	66.5 ± 6.5	66.3 ± 6.7	0.772a
Sex (male)	114 (88.4%)	107 (83.6%)	0.270
Comorbidities			
Diabetes mellitus	38 (29.5%)	42 (32.8%)	0.561
CAD	33 (25.6%)	34 (26.6%)	0.858
Hypertension	93 (72.1%)	95 (74.2%)	0.701
Surgical details			
Pre-dilation	120 (93.0%)	128 (100.0%)	0.007**
Preoperative stenosis (%)	77.4 ± 12.9	74.2 ± 12.0	**0.041a**
Post-dilation	61 (47.3%)	27 (21.1%)	**<0.001**
Residual stenosis (%)	20.0 (4.0, 26.3)	21.8 (11.6, 29.9)	**0.029b**
Symptomatic atherosclerotic stenosis	55 (42.6%)	61 (47.7%)	0.419
Periprocedural clinical event			
HD	2 (1.6%)	6 (4.7%)	0.276**
Stroke	1 (0.8%)	2 (1.6%)	0.995**
Any hemorrhage	4 (3.1%)	2 (1.6%)	0.687**
Follow-up event			
ISR	20 (15.5%)	12 (9.4%)	0.137
Symptomatic in-stent restenosis	9 (7.0%)	6 (4.7%)	0.434
Stroke	18 (14.0%)	16 (12.5%)	0.731
Ipsilateral stroke	5 (3.9%)	9 (7.0%)	0.265
Any hemorrhage	3 (2.3%)	1 (0.8%)	0.620**

Results are presented as numbers and percentages or as mean ± standard deviations or median (interquartile ranges). LDL, low-density lipoprotein cholesterol; BMI, body mass index; CAD, coronary heart disease; TIA, transient ischemic attack; HD, Hemodynamic depression; mRS, Modified Rankin Scale; ISR, in-stent restenosis; symptom-treatment: days from symptom to CAS procedure. The values with bold type represent statistically significant results with a *p*-value <0.05.

*Fisher exact test; **continuity correction; a. student’s *t*-test; b. Mann–Whitney U test.

Both CCS and OCS groups had comparable durations of imaging follow-up (*p* = 0.068) and ISR incidence rates (*p* = 0.137). Follow-up evaluations identified no significant differences between groups regarding stroke (*p* = 0.731), ipsilateral stroke (*p* = 0.265), or hemorrhage events (*p* = 0.620).

### 3.3 Factors associated with ISR

**Figure 2:**
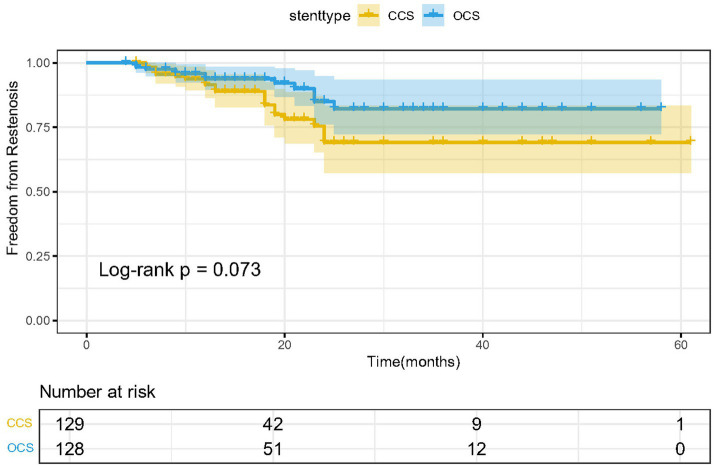
Comparison of survival estimates for restenosis between OCS and CCS.

In survival analysis, no difference was observed regarding restenosis between CCS and OCS (log-rank *p* = 0.073) (Figure 2). Preoperative baseline imbalances between the two groups might confound the comparison of restenosis rates. As the preoperative stenosis was significantly higher in the CCS group, we conducted an additional multivariate Cox regression. Further analysis using backward stepwise multivariate Cox regression, incorporating stent type and preoperative stenosis grade, showed that after adjustment, stent type (*p* = 0.134) remained non-significant, whereas preoperative stenosis grade (HR = 1.033, 95% CI: 1.003–1.063, *p* = 0.029) was a significant influencing factor.

Within the CCS subgroup, univariate analyses identified preoperative stenosis severity and symptomatic atherosclerotic stenosis as risk factors for ISR (Table 3). However, multivariate regression analysis confirmed symptomatic atherosclerotic stenosis as the only significant independent predictor (aHR = 2.669; 95% CI, 1.056–6.747; *p* = 0.038) (Table 4).

**Table 3:** Univariable COX analyses of factors associated with ISR in CCS and OCS.

Variable	CCS		OCS	
	HR (95%CI)	*p*	HR (95%CI)	*p*
Age (years)	0.969 (0.904–1.039)	0.374	1.076 (0.984–1.176)	0.108
Sex (male)	23.627 (0.037–15031.782)	0.337	0.809 (0.177–3.707)	0.785
BMI	1.042 (0.882–1.231)	0.630	0.844 (0.700–1.018)	0.076
LDL	0.976 (0.557–1.710)	0.931	0.798 (0.365–1.748)	0.573
Diabetes mellitus	1.140 (0.435–2.990)	0.790	3.641 (1.151–11.519)	**0.028**
Stroke history	0.945 (0.316–2.827)	0.919	2.773 (0.869–8.849)	0.085
CAD	0.730 (0.244–2.184)	0.573	2.500 (0.787–7.940)	0.120
Hypertension	2.172 (0.722–6.539)	0.168	0.476 (0.151–1.501)	0.205
Smoke	1.091 (0.454–2.626)	0.845	0.457 (0.123–1.691)	0.240
Onset style (stroke or TIA)	1.136 (0.378–3.416)	0.820	1.070 (0.233–4.915)	0.931
Side (left)	1.447 (0.577–3.628)	0.431	2.656 (0.715–9.859)	0.144
mRS	1.087 (0.679–1.739)	0.729	1.051 (0.556–1.987)	0.879
Pre-dilation	0.389 (0.114–1.330)	0.132	NA	NA
Preoperative stenosis	1.039 (1.002–1.078)	**0.036**	1.012 (0.966–1.061)	0.609
Stent length	1.050 (0.987–1.117)	0.122	NA	NA
Stent diameter	0.793 (0.504–1.250)	0.318	1.035 (0.308–3.481)	0.955
Post-dilation	1.256 (0.521–3.028)	0.611	1.707 (0.513–5.676)	0.383
Residual stenosis	1.002 (0.966–1.039)	0.929	1.041 (0.992–1.092)	0.102
Symptomatic atherosclerotic stenosis	2.884 (1.149–7.239)	**0.024**	0.706 (0.224–2.227)	0.552
Symptom-treatment	0.997 (0.990–1.004)	0.352	1.000 (0.998–1.002)	0.669

LDL, low-density lipoprotein cholesterol; BMI, body mass index; CAD, coronary heart disease; TIA, transient ischemic attack; HD, Hemodynamic depression; mRS, Modified Rankin Scale; ISR, in-stent restenosis; CCS, closed-cell stent; OCS, open-cell stent; symptom-treatment: days from symptom to CAS procedure. Results are presented as hazard ratio and 95% confidence intervals (CIs). The values with bold type represent statistically significant results with a *p*-value <0.05 in univariable analysis.

**Table 4:** Multivariable COX analyses of factors associated with ISR in CCS and OCS.

Variable	CCS		OCS	
	HR (95%CI)	*p*	HR (95%CI)	*p*
Diabetes mellitus			4.339 (1.362–13.821)	**0.013**
Preoperative stenosis	1.035 (0.999–1.072)	0.058		
Residual stenosis			1.046 (1.001–1.093)	**0.047**
Symptomatic atherosclerotic stenosis	2.669 (1.056–6.747)	**0.038**		

ISR, in-stent restenosis; CCS, closed-cell stent; OCS, open-cell stent. Results are presented as hazard ratio and 95% confidence intervals (CIs). The values with bold type represent statistically significant results with a *p*-value <0.05 in multivariable analysis.

Multivariable COX regression adjusted for preoperative stenosis and symptomatic atherosclerotic stenosis in CCS, while for age, BMI, diabetes mellitus, stroke history and residual stenosis in OCS.

Further subgroup analysis revealed a significantly higher ISR rate among symptomatic CCS-treated patients compared to asymptomatic patients (*p* = 0.028). Conversely, ISR rates between symptomatic and asymptomatic patients in the OCS subgroup showed no significant variation (*p* = 0.663) (Table 5).

**Table 5:** Comparison of ISR in symptomatic versus asymptomatic atherosclerotic stenosis in OCS and CCS.

Variable		OCS	*p*	CCS	*p*
Symptomatic atherosclerotic stenosis	ISR+	13 (23.6%)	**0.028**	5 (8.2%)	0.663
	ISR−	42 (76.4%)		56 (91.8%)	
Asymptomatic atherosclerotic stenosis	ISR+	7 (9.5%)		7 (10.4%)	
	ISR−	67 (90.5%)		60 (89.6%)	

Values in bold represent statistically significant results (*p* < 0.05).

In the OCS subgroup, BMI, diabetes mellitus, prior stroke history, and residual stenosis correlated with ISR on univariate analysis (Table 3). Subsequent multivariate analysis confirmed diabetes mellitus (*p* = 0.013) and residual stenosis severity (*p* = 0.047) as independent factors significantly associated with ISR (Table 4).

## 4 Discussion

Our study demonstrated that factors associated with ISR differ between CCS and OCS, despite similar overall incidences. In the CCS group, symptomatic atherosclerotic stenosis was identified as a significant risk factor for ISR. In the OCS group, residual stenosis and diabetes were significant factors.

CCS and OCS exhibit differences in radial support force, flexibility, and shear stress. CCS feature smaller mesh openings, reducing plaque protrusion. It is currently believed that CCS provide stronger radial support, reducing residual stenosis in conditions requiring robust support, such as calcification or vascular recoil; however, they demonstrate greater rigidity and poorer conformability. For vessels with significant curvature or angulation, open-cell stents (OCS) are favored due to their superior anatomical conformability, which aligns better with the native vessel course. OCS feature larger cell-free areas (9), offering weaker radial support but superior conformability. Although these studies did not explicitly compare stent types, given their mechanical properties, CCS, with greater rigidity, may reduce tortuosity and consequently lower restenosis risk.

### 4.1 Factors associated with ISR in CCS

Our findings showed an ISR rate consistent with previously reported rates of 5–11% (7). Symptomatic atherosclerotic stenosis was significantly associated with ISR in the CCS group. Stent selection is guided by patient and lesion characteristics to balance perioperative safety and restenosis risk. In this study, interventionalists favored CCS for patients with symptomatic atherosclerotic stenosis. Studies have indicated that surgical risks and complication rates differ between asymptomatic and symptomatic atherosclerotic stenosis patients (24). Symptomatic atherosclerotic stenosis may reflect more active and unstable lesions, which might explain our findings. Yoel Solomon et al. (39) reported that previously symptomatic status correlated with higher stroke and death rates compared with asymptomatic status. They attributed this finding to vulnerable atherosclerotic plaque characteristics associated with increased stroke risk. Another study also demonstrated a superior ISR-free rate in asymptomatic patients compared with symptomatic patients (25).

Previous studies have indicated that both preoperative and postoperative stenosis degrees are associated with increased restenosis risk (12, 17, 23). In our study, the CCS group had more frequent post-dilation due to perioperative safety considerations, resulting in lower residual stenosis. This finding reflects the mechanical characteristics of CCS, greater rigidity and reduced flexibility, which facilitate aggressive expansion (5, 26) and reduce restenosis risk. Consequently, preoperative stenosis influenced ISR more significantly than residual stenosis in the CCS group, even though this association was only marginally significant in our study (see Table 6).

**Table 6:** Univariable COX analyses of factors associated with ISR.

Variables	HR (95%CI)	*p*
Age (years)	1.013 (0.960–1.068)	0.644
Sex (male)	2.090 (0.499–8.753)	0.313
BMI	0.944 (0.834–1.068)	0.361
LDL	0.929 (0.589–1.466)	0.752
Diabetes mellitus	1.852 (0.912–3.760)	0.088
Stroke history	1.490 (0.688–3.228)	0.312
CAD	1.257 (0.581–2.721)	0.561
Hypertension	1.079 (0.499–2.335)	0.847
Smoke	0.845 (0.417–1.712)	0.640
Onset style (stroke or TIA)	1.080 (0.443–2.631)	0.866
Side (left)	1.806 (0.854–3.816)	0.122
mRS	1.115 (0.769–1.618)	0.565
Pre-dilation	0.291 (0.088–0.958)	**0.042**
Preoperative stenosis	1.033 (1.003–1.063)	**0.029**
Stent length	1.067 (1.001–1.137)	**0.046**
Stent diameter	1.090 (0.777–1.528)	0.617
Post-dilation	1.604 (0.797–3.226)	0.185
Residual stenosis	1.012 (0.985–1.040)	0.395
Symptomatic atherosclerotic stenosis	1.569 (0.780–3.516)	0.206
Symptom -treatment	0.999 (0.997–1.002)	0.597

LDL, low-density lipoprotein cholesterol; BMI, body mass index; CAD, coronary heart disease; TIA, transient ischemic attack; HD, hemodynamic depression; mRS, Modified Rankin Scale; ISR, in-stent restenosis; CCS, closed-cell stent; OCS, open-cell stent; symptom-treatment: days from symptom to CAS procedure. Results are presented as hazard ratio and 95% confidence intervals (CIs). The values with bold type represent statistically significant results with a *p*-value <0.05 in univariable analysis.

### 4.2 Factors associated with ISR in OCS

In the OCS group, BMI, diabetes, residual stenosis, and prior stroke were associated with ISR in univariate analysis. After multivariate adjustment, residual stenosis and diabetes remained significant. The impact of residual stenosis on restenosis has shown inconsistent results in different studies. Researches on extracranial carotid stenosis generally suggest that lower degrees of residual stenosis correlate with a lower incidence of restenosis. This study did not find a significant effect of residual stenosis in CCS, potentially attributable to better structural support provided by this type of stent.

CCS is characterized by higher radial force (9, 27) and greater rigidity, whereas OCS offers enhanced flexibility and conformability (5, 26). The OCS group exhibited higher residual stenosis, contributing notably to restenosis risk (9, 28). The relatively lower restenosis rate observed with OCS aligns with previous studies reporting this stent design as protective. Its inhibitory effect on ISR may be due to superior wall apposition and reduced plaque irritation (5, 13). Previous studies have suggested that OCS may be preferable for highly tortuous anatomy (10) and better conform to varying vessel diameters (13), demonstrating comparable efficacy in severe stenotic lesions. A 2024 study involving 46 carotid artery models reported that the distal slope and tortuosity significantly influenced adverse hemodynamic conditions, thereby increasing ISR risk; however, this study did not consider stent strut effects (29). Another study discussed elevated shear forces associated with CCS (7). Although intimal hyperplasia was more prevalent in the OCS group compared with CCS during follow-up, leading to progressive stenosis, this hyperplasia appeared benign (24) and did not significantly increase ISR rates. A randomized study previously reported lower ISR with OCS (5, 26), though it relied exclusively on ultrasound evaluation. Subsequent studies indicated that increased blood flow velocity following CAS due to stent placement might not correlate directly with ISR. The clinical advantages of OCS are particularly evident in tortuous vessels, where conformability mitigates restenosis risk. These physiological and methodological differences may explain conflicting conclusions across studies comparing stent designs. The distinct mechanical properties and context-dependent applications of CCS and OCS likely resulted in no significant difference in restenosis rates overall, as observed in our analysis, and help explain conflicting results in previous studies. These findings highlight the importance of refining techniques for CCS deployment and lesion-specific stent selection, optimizing lesion–stent matching, and ultimately reducing restenosis risk.

Diabetes is an established risk factor for restenosis, both in the entire cohort and specifically within the OCS group. Diabetes and smoking are independent risk factors for atherosclerotic stenosis and in-stent restenosis (30), with reports involving coronary and peripheral vascular stenosis. However, this association has not been consistently significant across studies, influenced by differences in study variables, patient populations, research centers, and sample size limitations (15, 24, 31). A study involving 70,453 coronary stent procedures found poor glycemic control was significantly associated with increased stent failure risk (31), attributed to prothrombotic and proinflammatory states linked to hyperglycemia, which accelerate atherosclerosis post-stenting. A meta-analysis of 20 randomized coronary trials also confirmed diabetes as significantly associated with higher restenosis risk (32), attributing this relationship to advanced glycation end products (AGEs) and insulin resistance. However, recent studies suggested that diabetes and plaque characteristics of restenosis may be related. Diabetes affects the pattern of plaque calcification (33), or presents diffuse narrowing located in smaller vessels (34). A study indicated that diabetes accelerates neointimal formation following vascular injury caused by interventional procedures through unique pathophysiological mechanisms. The extent of this effect may vary across different interventional approaches, though whether such differences actually exist requires further investigation (32). This suggests our findings may not solely stem from model limitations, but additional research is needed to confirm this relationship. When selecting stents, we considered plaque characteristics and vascular anatomy, but we lacked analysis of the relationship between these features and systemic factors such as diabetes. Larger contact area may intensify foreign-body reactions. Furthermore, inflammatory responses are associated with metabolic diseases like diabetes. The inflammatory response associated with metabolic disorders might therefore exert a stronger proliferative effect in the OCS group. In our study, OCS’s superior strut apposition may cause greater vascular wall irritation, potentially explaining the greater stenotic progression observed in patients with OCS, even if such progression does not meet the criteria for clinical restenosis. The absence of similar findings in the CCS group may be due to model limitations. And subgroup-specific ISR predictors might partly reflect confounding introduced by anatomy-driven stent selection. The CCS group showed a different pattern and demonstrated lower residual stenosis due to more frequent post-dilation, making it less prone to clinically significant restenosis with comparable levels of stenotic progression.

### 4.3 Limitations

This study has several limitations. First, the relatively small sample size from a single hospital limits the generalizability of our conclusions, and the low incidence of ISR may have limited the detection of statistically significant differences. Second, although we stated that CCS were preferred for symptomatic stenosis in the institution, there was no statistically difference in symptomatic stenosis between CCS and OCS groups. Even if clinically driven, the selection bias can still confound ISR comparisons.

Furthermore, this study only measured the degree of stenosis without assessing lesion and vessel characteristics such as plaque vulnerability including ulceration on angiography or ultrasound, the vessel curvature or angulation on angiography, or stent landing zones. Longer and more tortuous lesions limit stent navigation and reduce adequate apposition (35). Regarding shear stress, studies indicate that regular blood flow generates higher wall shear stress, which inhibits intimal hyperplasia (36). Low wall shear stress (WSS) induced by flow turbulence is considered a major cause of restenosis. One study discussed how geometric changes in the carotid artery following CAS may lead to altered hemodynamics, noting that increased tortuosity of residual stenotic segments produces more pronounced turbulent flow during peak systole, thereby increasing ISR risk (29). Extensive calcification (37), the distance from the stent implantation site to bifurcations and post-implantation geometric alterations (38) are also associated with restenosis. The absence of these anatomical variables in our dataset limits the ability to fully interpret the differences in ISR between CCS and OCS.

Future research should incorporate randomized controlled trials and further investigate hemodynamic impacts on restenosis between stent types.

## 5 Conclusion

Our study observed a non-significant trend toward higher ISR in the CCS group compared with the OCS group prior to adjustment, in patients with moderate symptomatic stenosis or severe asymptomatic stenosis. Multivariate analysis identified differences in independent risk factors for ISR between CCS and OCS. Symptomatic atherosclerotic stenosis was critical for CCS, while diabetes and residual stenosis severity were key factors associated with ISR for OCS.

## Data Availability

The raw data supporting the conclusions of this article will be made available by the authors, without undue reservation.
